# Integrated Hypothalamic Transcriptome Profiling Reveals the Reproductive Roles of mRNAs and miRNAs in Sheep

**DOI:** 10.3389/fgene.2019.01296

**Published:** 2020-01-15

**Authors:** Zhuangbiao Zhang, Jishun Tang, Ran Di, Qiuyue Liu, Xiangyu Wang, Shangquan Gan, Xiaosheng Zhang, Jinlong Zhang, Mingxing Chu, Wenping Hu

**Affiliations:** ^1^ Key Laboratory of Animal Genetics and Breeding and Reproduction of Ministry of Agriculture and Rural Affairs, Institute of Animal Science, Chinese Academy of Agricultural Sciences, Beijing, China; ^2^ Institute of Animal Husbandry and Veterinary Medicine, Anhui Academy of Agricultural Sciences, Hefei, China; ^3^ State Key Laboratory of Sheep Genetic Improvement and Healthy Production, Xinjiang Academy of Agricultural and Reclamation Sciences, Shihezi, China; ^4^ Tianjin Institute of Animal Sciences, Tianjin, China

**Keywords:** hypothalamus, mRNAs, miRNAs, GnRH, reproduction, sheep

## Abstract

Early studies have provided a wealth of information on the functions of microRNAs (miRNAs). However, less is known regarding their functions in the hypothalamus involved in sheep reproduction. To explore the potential roles of hypothalamic messenger RNAs (mRNAs) and miRNAs in sheep without FecB mutation, in total, 172 and 235 differentially expressed genes (DEGs) and 42 and 79 differentially expressed miRNAs (DE miRNAs) were identified in polytocous sheep in the follicular phase versus monotocous sheep in the follicular phase (PF vs. MF) and polytocous sheep in the luteal phase versus monotocous sheep in the luteal phase (PL vs. ML), respectively, using RNA sequencing. We also identified several key mRNAs (e.g., *POMC*, *GNRH1*, *PRL*, *GH*, *TRH*, and *TTR*) and mRNA–miRNAs pairs (e.g., *TRH* co-regulated by oar-miR-379-5p, oar-miR-30b, oar-miR-152, oar-miR-495-3p, oar-miR-143, oar-miR-106b, oar-miR-218a, oar-miR-148a, and *PRL* regulated by oar-miR-432) through functional enrichment analysis, and the identified mRNAs and miRNAs may function, conceivably, by influencing gonadotropin-releasing hormone (GnRH) activities and nerve cell survival associated with reproductive hormone release *via* direct and indirect ways. This study represents an integral analysis between mRNAs and miRNAs in sheep hypothalamus and provides a valuable resource for elucidating sheep prolificacy.

## Introduction

Reproduction, one of the major factors significantly affecting the sheep industry, is a complicated but important physiological process. The success of reproduction is mainly dependent on the release of hormones, including gonadotropin-releasing hormone (GnRH) released from the hypothalamus, follicle-stimulating hormone (FSH) and luteinizing hormone (LH), which are both secreted from the pituitary (Cao et al., 2018a). Following the release of hormones, a series of events associated with reproduction, such as ovulation and fertilization, could occur.

It is well known that reproductive traits, such as litter size, are controlled by minor polygene. Researchers have found several major fecundity genes which considerably influence sheep prolificacy, such as bone morphogenetic protein receptor IB (*BMPRIB*), bone morphogenetic protein 15 (*BMP15*) (Chu et al., 2007), and growth differentiation factor 9 (*GDF9*) (Chu et al., 2011). FecB is a mutation in *BMPRIB* occurring in base 746 from A to G. This base change further results in changes in protein function due to a key amino acid transition from glutamine to arginine (Fogarty, 2009). Sheep with one copy of the FecB mutation can experience significant increase in litter size, by 0.67, while this increase is about 1.5 when there are two mutated copies (Liu et al., 2014). Moreover, this mutation was also detected in diverse sheep species, such as Booroola Merino sheep (Mulsant et al., 2001) (Australia), Garole sheep (Polley et al., 2010) (India), Hu sheep (Davis et al., 2006) (China), and Small Tail Han sheep (STH sheep; China) (Davis et al., 2006). STH sheep, an indigenous species in China, has attracted much attention for its excellent traits (Liu et al., 2016; Chao et al., 2017), especially the higher prolificacy (Davis et al., 2006). Furthermore, STH sheep can be divided into three genotypes based on the effects of FecB mutation, better known as FecB BB (with two-copy FecB mutations), FecB B^+^ (with one-copy FecB mutation), and FecB^++^ (with no FecB mutation). Usually, compared to sheep with the other two genotypes, STH sheep with FecB^++^ show a monotocous phenomenon. However, the fact is that there are STH sheep with FecB^++^ and which show a polytocous phenomenon (Davis et al., 2006), and how this mechanism was established remains largely unclear.

With advances in sequencing, the application of RNA sequencing (RNA-seq) in animals, including sheep (Jiang et al., 2014; Zhang et al., 2019a; Zhang et al., 2019b), mice (Beck et al., 2018), and cattle (Correia et al., 2018), enables integral analysis of the expression profiling of mRNA and miRNAs. Therefore, RNA-seq has been widely used to understand some complex traits. Regarding the generation of miRNA, precursor miRNA is transcribed mainly by RNA polymerase II, then processed into mature miRNA (Gebert and Macrae, 2019). Significantly, miRNAs play pivotal roles in life processes, such as muscle growth (Cao et al., 2018c), fleece and hair development (Liu et al., 2018), and neural development (Schratt et al., 2006). Additionally, reproduction is an extremely complex process, and the use of RNA-seq may contribute to enhancing our understanding of sheep fecundity. By comparing the mRNA and miRNA expression patterns in European mouflon and sheep, a research (Yang et al., 2018) found several key mRNAs, such as *INHBA*, *SPP1*, and *ZP2*, and miRNAs, such as miR-374a and miR-9-5p, which may be responsible for the success of female sheep reproduction. Pokharel et al. (2018) detected and characterized some key miRNAs and mRNAs in sheep ovary which may be responsible for sheep prolificacy. Thereby, the identification and functional analysis of mRNAs and miRNAs and characterization of their mutual interaction through sequencing technology may provide new insights into the prolific mechanism in STH sheep with the FecB^++^ genotype, which has so far been difficult to elucidate using standard approaches.

Therefore, in the present study, we applied transcriptomics analysis in PF vs. MF and PL vs. ML to identify DEGs and DE miRNAs and analyze their potential functions, expecting to elucidate the potential prolific mechanism in sheep with the FecB^++^ genotype and act as a reference for other female mammals.

## Material and Methods

### Preparation of Animals

First, the TaqMan probe (Liu et al., 2017) was applied to genotype the sheep population (*n* = 890). Then, 12 sheep with no significant differences in sheep age, weight, height, body length, chest circumference, and tube circumference were selected from 142 STH sheep with the FecB^++^ genotype and grouped into the polytocous group (*n* = 6, litter size ≥2) and monotocous group (*n* = 6, litter size = 1) according to their litter size records. Additionally, all the sheep were bred under the same conditions, with free access to water and feed, in a sheep farm of the Tianjin Institute of Animal Sciences.

All selected sheep were processed by estrus synchronization with Controlled Internal Drug Releasing Device (CIDR; progesterone 300 mg; Zoetis Australia Pty. Ltd., NSW, Australia) for 12 days. The six sheep, comprising three polytocous sheep and three monotocous sheep, were slaughtered within 45–48 h after CIDR removal (follicular phase), the remaining six sheep were slaughtered on day 9 after CIDR removal (luteal phase). Finally, the selected sheep were divided into four groups, including polytocous sheep in the follicular phase (PF), polytocous sheep in the luteal phase (PL), monotocous sheep in the follicular phase (MF), and monotocous sheep in the luteal phase (ML), on the basis of their littering record and estrous cycle.

### Preparation of Tissues, RNA Extraction, and Sequencing

Hypothalamic tissues were collected from 12 killed sheep and immediately stored at −80°C until being used. Then, total RNA was isolated using TRIzol Reagent (Invitrogen, Carlsbad, CA, USA) under the manufacturer’s instructions, and the quality and integrity of isolated RNA were assessed by an Agilent 2100 Bioanalyzer (Agilent Technologies, CA, USA) and electrophoresis. The high-quality RNA of 3 μg of each sample was used to build the mRNA library using a NEBNext Ultra Directional RNA Library Prep Kit for Illumina (NEB, Ipswich, USA), which has been described in our previous work (Zhang et al., 2019b). All the sequencing works were conducted in Annoroad Gene Technology Co., Ltd. (Beijing, China).

The fragments with lengths of 18–30 nt, which were obtained from total RNA through the gel separation technique, were used as templates to synthesize the first strand of complementary DNA (cDNA). The second strand of cDNA was also synthesized in the presence of deoxynucleoside triphosphates (dNTPs), ribonuclease H, and DNA polymerase I. Then the obtained double-stranded cDNA was processed with end-repair, the addition of base A and sequencing adaptors, and uracil-*N*-glycosylase (UNG) enzyme digestion. Finally, polymerase chain reaction was conducted to build the miRNA library.

In addition, a paired-end sequencing approach for mRNAs and miRNAs was conducted using an Illumina HiSeq 2500.

### Quality Control, Mapping and Assembly

Raw reads were filtered using in-house software of fqtools_plus-v2.0.0 according to strict criteria, including removing reads with adaptor contaminants, low-quality reads, and reads with N bases accounting for more than 5%. Then, HiSAT2 (Kim et al., 2015) was used to map the cleaned reads to the reference genome (Oarv3.1), and both the sheep reference genome and genome annotation file were downloaded from ENSEMBL (http://www.ensembl.org/index.html). Subsequently, StringTie 1.3.2d (Pertea et al., 2015) was used to assemble transcripts of mRNAs.

Several criteria were also implemented to generate clean miRNA reads, including removing reads without a 3′ adapter, reads without insert fragment, reads with lengths beyond the normal range, raw reads containing too much A/T, and some low-quality reads using in-house scripts. Furthermore, the cleaned data of miRNA were matched against the sheep reference genome (Oarv3.1) by Bowtie v1.1.2 (Langmead et al., 2009).

### Differential Expression and Functional Enrichment Analysis of mRNAs

To validate the expression level of mRNAs, the fragments per kilobase per million mapped reads (FPKM) values (Trapnell et al., 2010) were calculated to represent the gene expression level, and DESeq 2-1.4.5 (Wang et al., 2010) was also used to detect the DEGs between two comparisons based on FPKM values. Additionally, a gene with fold change >1.5 and *p* < 0.05 was considered as a DEG in PF vs. MF and PL vs. ML. In addition, we also performed Gene Ontology (GO) and Kyoto Encyclopedia of Genes and Genomes (KEGG) enrichment analysis. We first downloaded the Uniprot database, where each sequence contains the GO annotation and KEGG annotation species (sheep) of the sequence as well as gene and protein names. All genes of sheep to be analyzed were compared with the Uniprot database by blast (NCBI-blast 2.2.28) to find the best alignment result for each sequence, and corresponding to GO and KEGG annotation results. Then, we also downloaded the corresponding relationship between the entry name and number provided on the websites of GO and KEGG, as well as the classification hierarchy file, and summarize the GO and KEGG classification of the genes we obtained. Lastly, a particular GO term or KEGG pathway with a hypergeometric *p* value < 0.05 was thought to indicate significant enrichment.

### Differential Expression Analysis and Prediction of Target Genes of miRNAs

The miRDeep v2.0.0.8 (Friedländer et al., 2012) was applied to identify the known and novel miRNAs by mapping clean reads and hairpins to mature miRNAs recorded in the miRbase database (Griffiths-Jones, 2006). In addition, transcripts per million (TPM) were calculated to represent miRNA expression levels on the basis of the reads number. DESeq2-1.4.5 (Wang et al., 2010) was also applied to identify DE miRNAs in PF vs. MF and PL vs. ML, and the threshold of fold change >1.5, *p* < 0.05** was considered to indicate differential expression. Furthermore, miRanda v3.3a (Enright et al., 2004) was used to predict the target genes of miRNAs.

### Integral miRNA–mRNA Networks Analysis

To precisely identify key DE miRNAs and DEGs associated with reproduction, a network containing DE miRNAs and DE mRNAs, on the basis of miRNA functions (Gebert and Macrae, 2019), was built using Cytoscape_v3.5.0 (Shannon et al., 2003), and only mRNAs exhibiting negative relationship with miRNAs were included in miRNA–mRNA interaction networks.

### Data Validation

In order to validate the accuracy of sequencing data, four DEGs, including *CRH*, *FOXG1*, *TTR*, and *POMC*, and four DE miRNAs, including oar-miR-433-3p, oar-miR-495-3p, oar-miRNA-16b, and oar-miR-143, were selected for data validation. First, the primers of DEGs and DE miRNAs were synthesized by Beijing Tianyi Huiyuan Biotechnology Co., Ltd. (Beijing, China) (**Supplementary Table 1**) for subsequent reverse transcription, which was performed using PrimeScript™ RT reagent kit (TaKaRa) for mRNAs and miRcute Plus miRNAs First-Strand cDNA Kit (TIANGEN, Beijing, China) for miRNA. Furthermore, quantitative PCR (qPCR) was conducted with the SYBR Green qPCR Mix (TaKaRa, Dalian, China) for mRNAs and miRcute Plus miRNA qPCR Kit (TIANGEN, Beijing, China) for miRNAs using a RocheLight Cycler^®^480 II system (Roche Applied Science, Mannheim, Germany). In addition, β-actin (for mRNA) and U6 small nuclear RNA (snRNA; for miRNA) were utilized as reference gene/miRNA to calculate the relative expression level with the method of 2^-ΔΔct^ (Livak and Schmittgen, 2001). The qPCR for mRNAs was conducted in the following procedure: initial denaturation at 95°C for 5 minutes, followed by 40 cycles of denaturation at 95°C for 5 s, then annealing at 60°C for 30 s. While the qPCR for miRNA was conducted in the following procedure: initial denaturation at 95°C for 15 minutes, followed by 40 cycles of denaturation at 94°C for 20 s, then annealing at 60°C for 34 s. All the qPCR results were presented as the mean ± SD.

## Results

### mRNA and miRNA Profiling

**Figure 1:**
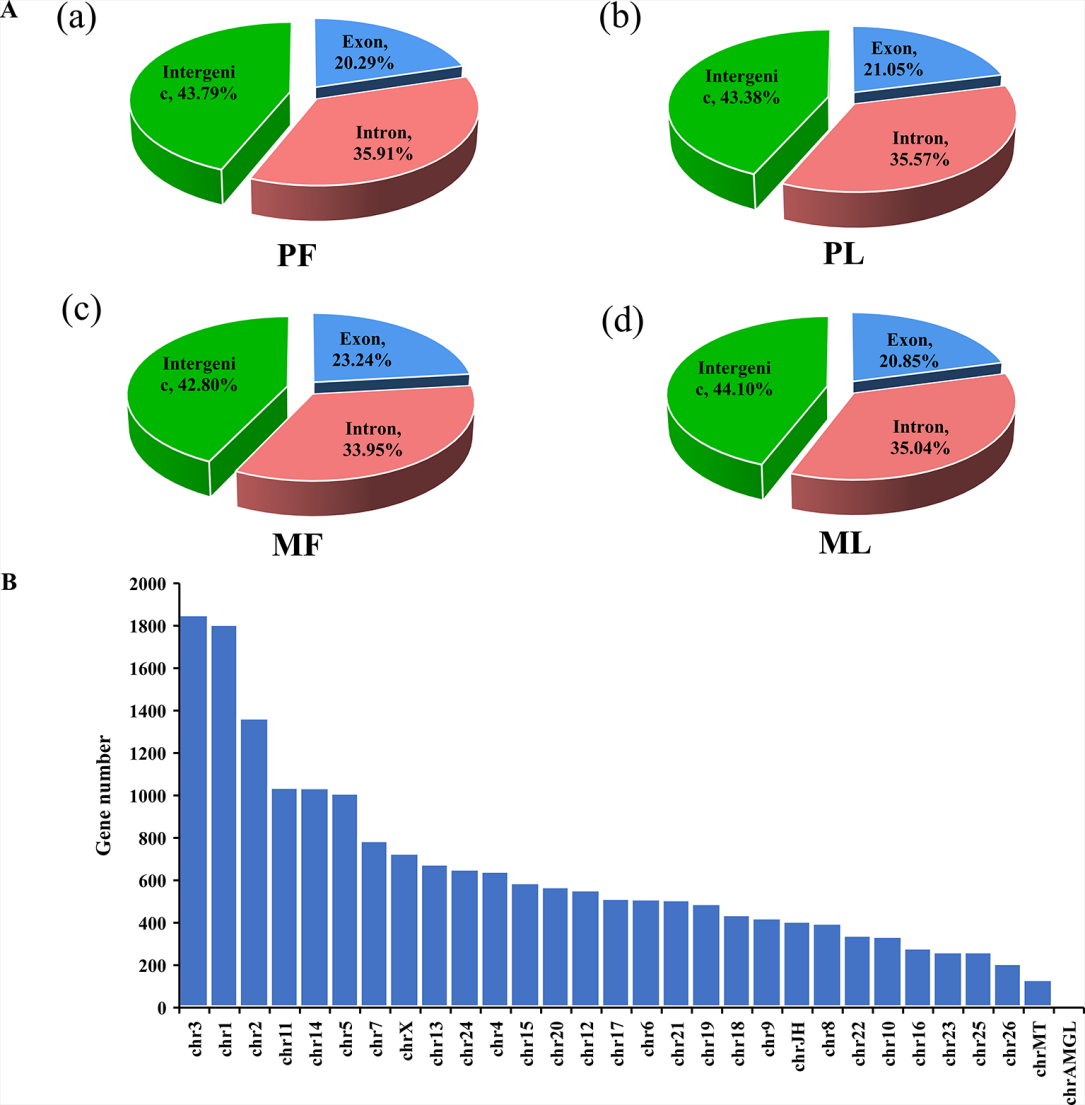
Mapping region and chromosome distribution of identified mRNAs. **(A)** Mapping region of identified genes at the reference genome in polytocous sheep in the follicular phase (PF) (**a**), polytocous sheep in the luteal phase (PL) (**b**), monotocous sheep in the follicular phase (MF) (**c**), and monotocous sheep in the luteal phase (ML) (**d**). **(B)** Chromosome distribution of identified genes from the hypothalami in the PF, MF, PL, and ML.

To fully characterize the globally hypothalamic mRNA and miRNA expression differences between sheep with the same genotype but different litter sizes, RNA-seq was used to detect their expression profile in the hypothalamus. In total, RNA-seq for mRNA generated approximately 1,519 million raw reads and 1,460 million clean reads (**Supplementary Table 2**) after data filtering. Overall, 21,221 mRNAs were identified (**Supplementary Table 3**) after mapping to sheep genome, and our results also suggested that many mRNAs were located in the intergenic region (nearly 45%), followed by the intron (about 35%) and exon (more than 20%) regions (**Figure 1A** and **Supplementary Table 4**).

**Figure 2:**
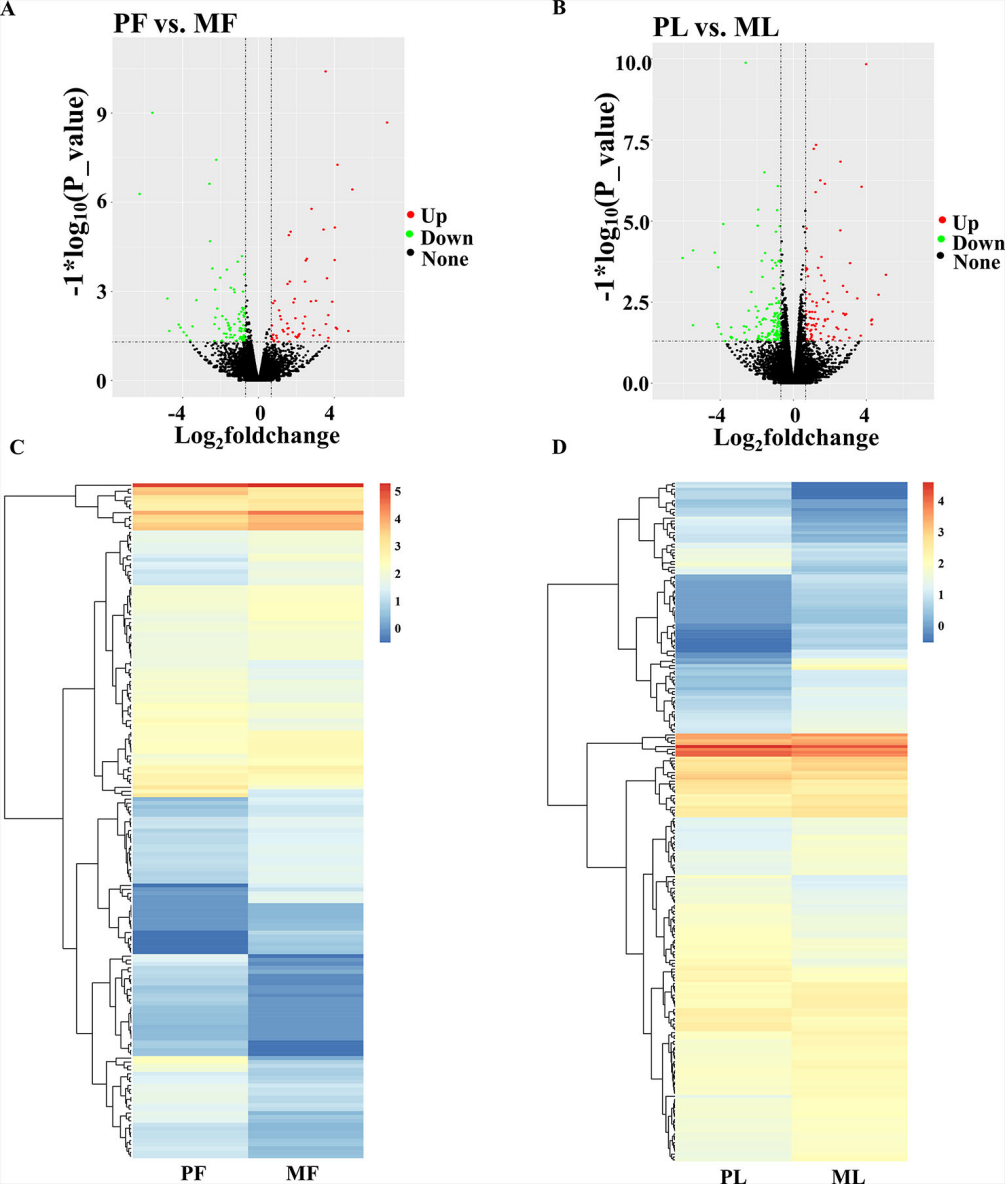
Differentially expressed genes (DEGs) analysis. **(A)** Volcano plot of identified genes in PF vs. MF, where *red* and *green* represent up- or downregulation, respectively, same below. **(B)** Volcano plot of identified genes in PL vs. ML. **(C)** Heat maps showing the expression intensity of 794 DEGs in the follicular phase, including PF and MF. **(D)** Heat maps showing the expression intensity of 1,044 DEGs in the luteal phase, including PL and ML.

Regarding the expression level of mRNAs, our results showed that the FPKM of those genes obtained from RNA-seq at <50 constituted nearly 90%, and the high-expression genes, i.e., those with FPKM >500, constituted about 0.5% (**Supplementary Table 3**), which suggested that the data obtained from the hypothalamus *via* RNA-seq were relatively reasonable. Furthermore, the chromosome distribution of mRNAs indicated that chromosome 3 contains 9.79% of the genes identified from the hypothalamus, followed by chromosome 1 (9.55%) and chromosome 2 (7.22%) (**Figure 1B** and **Supplementary Table 5**). Additionally, the number of DEGs identified from PF vs. MF (**Figure 2A** and **Supplementary Table 6**) and PL vs. ML (**Figure 2B** and **Supplementary Table 6**) were 172 and 235, respectively. Among these DEGs, 79 and 90 were upregulated, while 93 and 145 were downregulated in PF vs. MF and PL vs. ML, respectively. In addition, the expression density of DEGs displayed obviously different expression patterns between PF and MF, and between PL and ML (**Figures 2C**, **D**).

**Figure 3:**
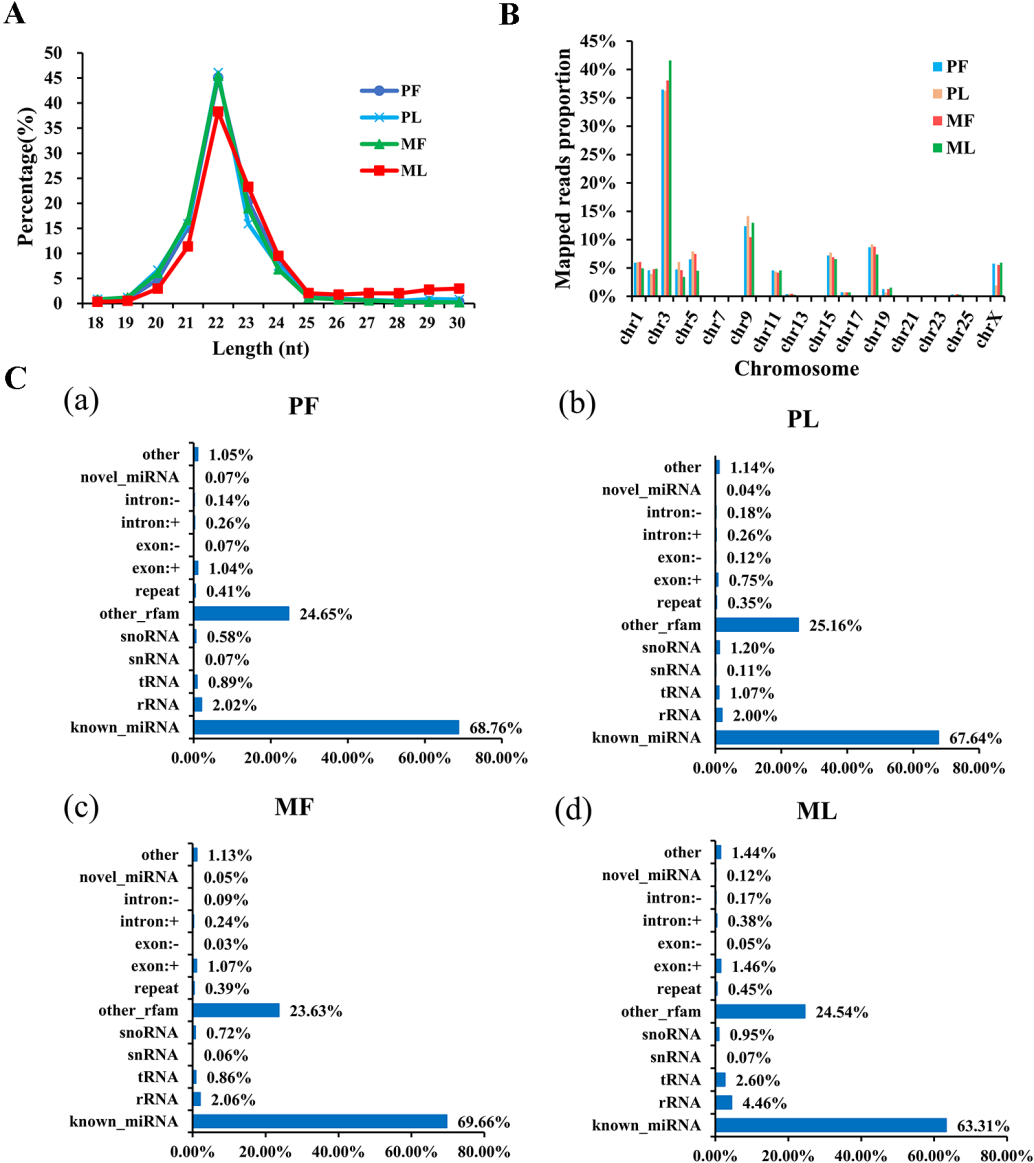
Characterization of microRNA (miRNA) profiling and the percentage of detected miRNAs from ncRNAs. **(A)** Length distribution of clean reads from identified miRNA fragments. **(B)** The chromosome distribution of identified miRNAs from hypothalami. **(C)** Categories of identified non-coding RNAs (ncRNAs) *via* sequencing in PF (**a**), PL (**b**), MF (**c**), and ML (**d**).

Regarding miRNAs, RNA-seq generated approximately 315 million raw reads and 267 million clean reads (**Supplementary Table 7**) with lengths ranging from 18 to 30 nt (**Figure 3A**) after removing low-quality reads. Overall, 623 miRNAs were detected (**Supplementary Table 8**). In addition, the chromosome distribution of identified miRNAs was also determined. As **Figure 3B** shows, the chromosome distribution of miRNAs from 1 to *X* varies (**Supplementary Table 9**), and most of the identified miRNAs were located at chromosome 3 (nearly 40%), followed by chromosome 9 (nearly 15%) and chromosome 18 (nearly 9%). Interestingly, chromosome 3 also contains the most mRNAs (**Figure 3B**). Also, a diversity of non-coding RNAs (ncRNAs), including transfer RNAs (tRNAs), snRNAs, miRNAs, etc., were also identified (**Figure 3C** and **Supplementary Table 10**), and the known miRNAs account only for a small part of all the identified ncRNAs. In addition, the target genes of miRNAs in PF vs. MF and PL vs. ML were predicted to be 1,611 and 2,120, respectively (**Supplementary Table 11**).

**Figure 4:**
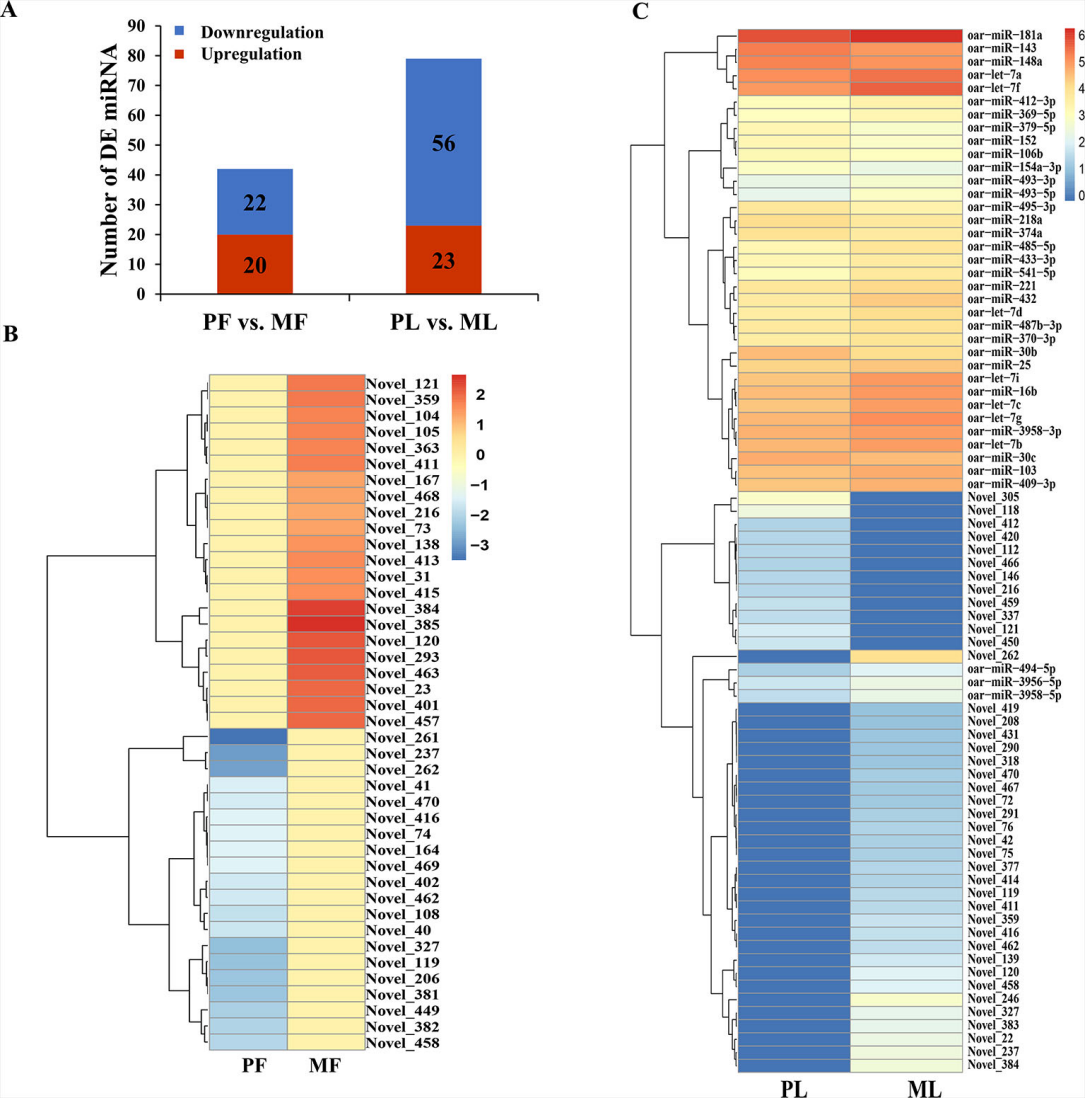
Differentially expressed (DE) microRNA (miRNA) analysis. **(A)** DE miRNAs in PF vs. MF and PL vs. ML. Heat maps showing the expression intensity of 42 and 79 DE miRNAs in the follicular phase including PF and MF **(B)** and the luteal phase including PL and ML **(C)**, the names of miRNAs were also labeled.

Additionally, the DE miRNAs identified from PF vs. MF and PL vs. ML were 42 and 79, respectively. Of these DE miRNAs, 20 and 23 were upregulated, while 22 and 56 were downregulated, respectively (**Figure 4A** and **Supplementary Table 12**). In addition, the expression density of DEGs displayed obviously different expression patterns between PF and MF, and between PL and ML (**Figures 4B, C**).

### GO and KEGG Enrichment Analysis of DEGs

**Figure 5:**
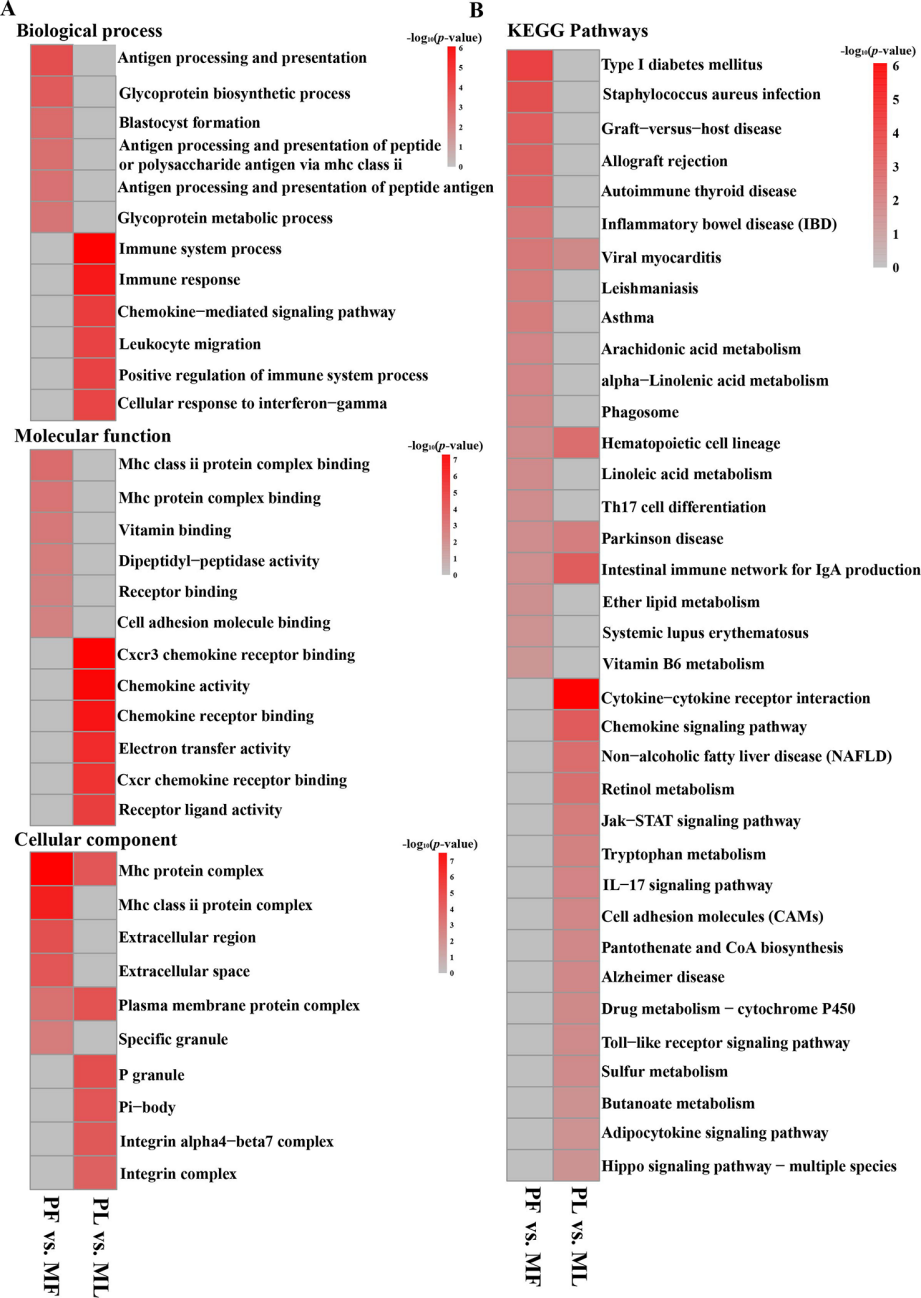
Functional enrichment analysis of DEGs. **(A)** Top enriched GO terms at the biological process, molecular function, and cellular component level in PF vs. MF and PL vs. ML, in addition, the gray represents no enrichment, same below. **(B)** Top enriched KEGG pathways in PF vs. MF and PL vs. ML.

To better understand the potential functions of the DEGs, GO term and KEGG pathway analyses were performed. In GO analysis, the most enriched term in PF vs. MF was the MHC protein complex (GO:0042611). Other GO terms related to the MHC protein were also enriched, such as MHC class II protein complex binding (GO:0023026) and MHC protein complex binding (GO:0023023), indicating the crucial role of the MHC protein in the hypothalamic functions (**Figure 5A** and **Supplementary Table 13**). Regarding PL vs. ML, the top 2 enriched terms were the immune system process (GO:0002376) and immune response (GO:0006955). In addition, some GO terms associated with chemokine receptors, including CXCR3 chemokine receptor binding(GO:0048248) and chemokine receptor binding (GO:0042379), were also highly enriched, suggesting the important roles of the immune system and chemokine receptors in the hypothalamus at the luteal phase (**Figure 5A** and **Supplementary Table 13**).

KEGG analysis in PF vs. MF (**Figure 5B** and **Supplementary Table 14**) showed that the most enriched pathway was type I diabetes mellitus (map04940). In addition, other metabolic pathways, such as alpha-linolenic acid metabolism (map00592) and arachidonic acid metabolism(map00590), were also enriched. Regarding PL vs. ML, the top enriched pathways were cytokine–cytokine receptor interaction (map04060). A pathway named the Jak-STAT signaling pathway (map04630), which has been found to participate in the reproductive process (Ko et al., 2018), was also enriched.

### Analysis of Integrated miRNA–mRNA Co-Expression Network

**Figure 6:**
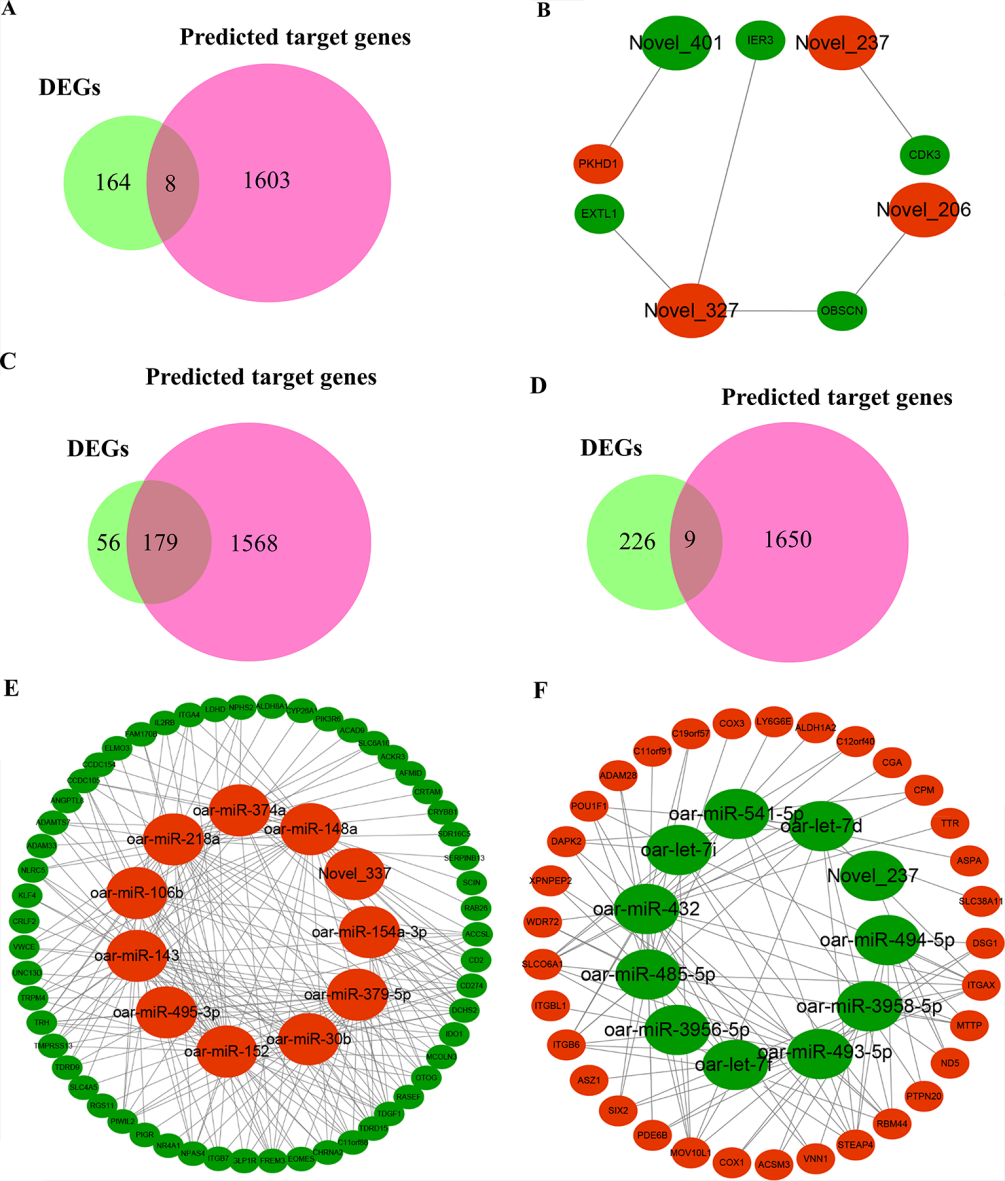
Overview of mRNA–miRNA networks. **(A)** Overlapped genes in PF vs. MF between DEGs and miRNA-targeted genes. **(B)** Hypothalamus network in PF vs. MF of four miRNAs containing three upregulated miRNAs and one downregulated miRNA and five target genes, where *large* and *small ellipses* represent miRNAs and DEGs, respectively, in addition, *red* and *green* represent up- or downregulation, respectively; same below. **(C)** Overlapped genes in PL vs. ML between DEGs and predicted target genes negatively modulated by known miRNAs. **(D)** Overlapped genes in PL vs. ML between DEGs and novel miRNA-targeted genes. **(E)** Main hypothalamic upregulated network in PL vs. ML containing the top 10 upregulated miRNAs and one novel miRNA and 55 target genes. **(F)** Main hypothalamic downregulated network in PL vs. ML containing the top 10 downregulated miRNAs and one novel miRNA and 33 target genes.

To fully understand the potential reproductive roles of miRNAs, we built interactome networks using DE miRNAs and their targets (DEGs). In total, 42 DE miRNAs (novel miRNAs) in PF vs. MF were predicted to target 1,611 genes (**Supplementary Table 15**). The number of overlapped genes, which means the target genes were also DEGs, was 8 (**Figure 6A** and **Supplementary Table 16**). An mRNA–miRNA co-expression network was then constructed, where 5 DEGs were targeted by 3 novel miRNAs (**Figure 6B**). Regarding PL vs. ML, 38 known and 41 novel DE miRNAs were predicted to target 1,747 and 1,659 genes (**Supplementary Table 15**), and the numbers of overlapped genes were 179 and 9, respectively (**Figures 6C, D** and **Supplementary Table 16**). The main upregulated miRNA–mRNA co-expression network suggested that 55 DEGs were targeted by 11 DE miRNAs containing the top 10 upregulated known miRNAs and one novel miRNA (**Figure 6E**). The main downregulated miRNA–mRNA co-expression network suggested that 33 DEGs were targeted by 11 DE miRNAs containing the top 10 downregulated known miRNAs and one novel miRNA (**Figure 6F**).

### Data Validation

**Figure 7:**
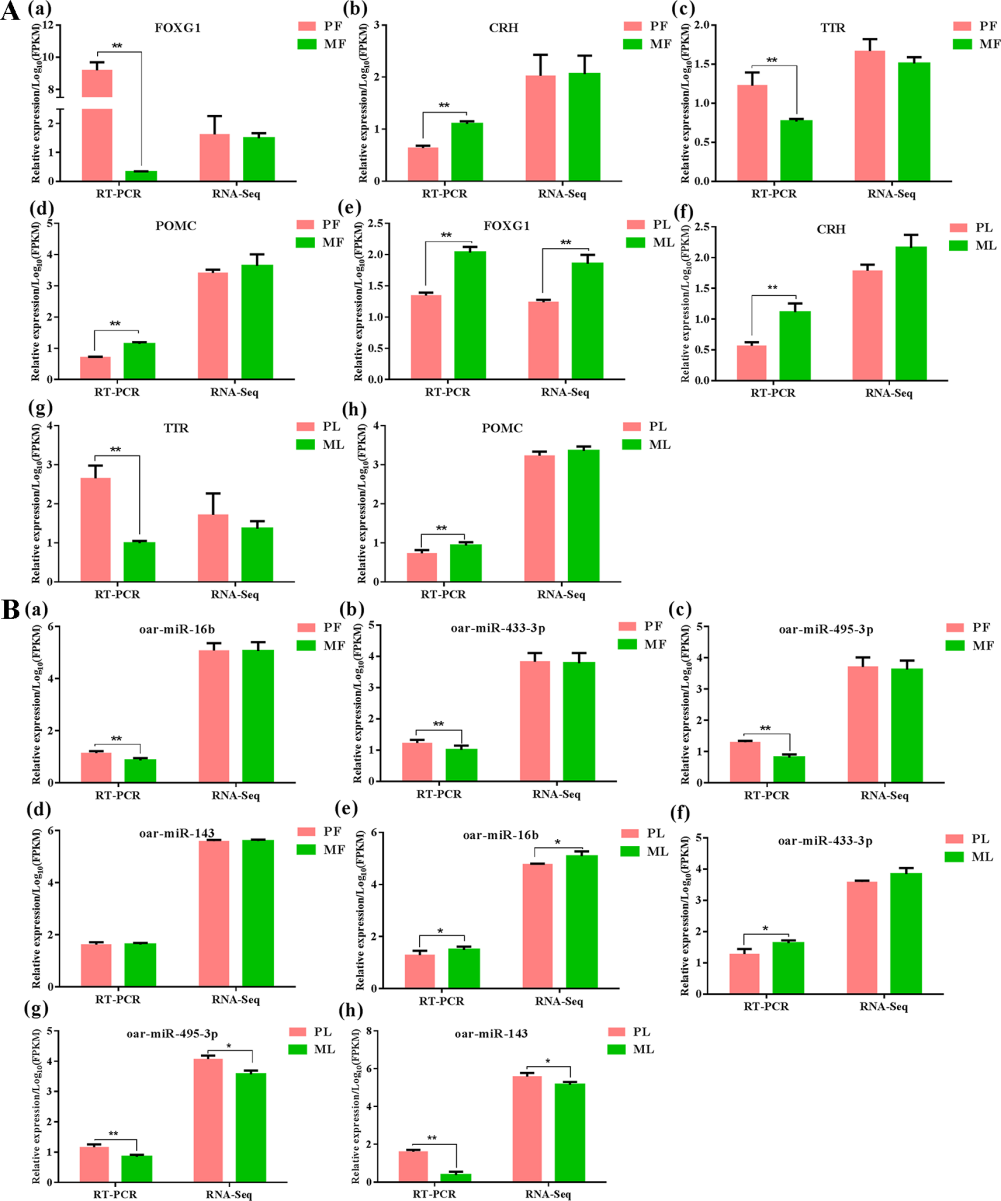
Data validation of mRNAs **(A)** and miRNAs **(B)** by qPCR in PF, PL, MF and ML, meanwhile ** represents p < 0.01, while * represents p < 0.05. *FOXG1*: Forkhead box L1, *CRH*: corticotropin-releasing hormone, *TTR*: transthyretin, *POMC* proopiomelanocortin.

>In order to assess the accuracy of sequencing, qPCR was applied to verify the RNA-seq data. The results indicated that both mRNAs and miRNAs in sheep hypothalamus displayed expression patterns similar to the sequencing results (**Figure 7**), demonstrating the reliability of the data generated from RNA-seq.

## Discussion

In this study, we initially identified 172 and 235 DEGs, and 42 and 79 DE miRNAs in two comparisons (PF vs. MF and PL vs. ML) through RNA-seq. Of these DE miRNAs, miRNA family members including the let-7 and oar-miRNA-200 family exhibited differential expression levels. Furthermore, one study detecting 48 DE miRNAs from sheep ovary, including the let-7 and oar-miRNA-200 family members, suggested that those identified miRNAs were differentially expressed in seasonal and non-seasonal sheep breeds (Zhai et al., 2018). Therefore, some miRNAs, such as let-7 and oar-miRNA-200 family members may not be only species-specific but also phase- or fecundity-specific in sheep. In addition, some miRNAs, including miRNA-138 and miRNA-212, were detected in rat hypothalamus (Amar et al., 2012), which differed significantly from miRNAs identified in sheep hypothalamus (both miRNA-138 and miRNA-212 in our results failed to be detected). Besides, several miRNAs, such as miRNA-200 family members, were conserved in the hypothalamus of mice (Choi et al., 2008; Crépin et al., 2014), rat (Sangiaoalvarellos et al., 2014), and zebrafish (Garaffo et al., 2015), as well as sheep (our results). In summary, we confirmed that several miRNAs are conserved in many animals, but there were also miRNAs that showed a species-specific distribution in the hypothalamus, which means those differences may be responsible for the differences between sheep and rats, and even other non-mammals.

### Functional Analysis of DEGs in PF vs. MF

In the functional enrichment analysis of DEGs in PF vs. MF, several key genes, including prolactin (*PRL*), proopiomelanocortin (*POMC*), and gonadotropin releasing hormone 1 (*GNRH1*), were found to participate in the reproductive process. Some researchers have proven that PRL and E2 could respond rapidly to stimulation in the arcuate nucleus (ARC) of rat hypothalamic slices (Nishihara and Kimura, 1989). Araujo-Lopes et al. (2014) revealed that PRL could regulate the activities of GnRH through modulating kisspeptin neurons in the ARC of female rats and inhibit LH secretion, causing a series of alterations in the estrous cycle. Our results indicated that the expression of *PRL* in PF was more than three times that of PRL in MF. Therefore, coupled with the inhibitory role of PRL on LH, we speculate that *PRL* may affect LH or FSH activities by influencing the pulsatile GnRH wave in the hypothalamus.

POMC neurons, as a key upstream factor affecting hypothalamic hormone release, were found to be sensitive to metabolic hormones such as leptin (Wilson and Enriori, 2015) and enhance kisspeptin neuron activities in rodents, resulting in increased GnRH secretion (Muroi and Ishii, 2016). Leptin can act in the hypothalamus directly, eliciting the release of GnRH (Guzmán et al., 2019), and promoting the expression of *POMC* (Perello et al., 2007). Although the stimulatory effects of POMC on kisspeptin have been known for a long time, how this signaling is established remains poorly understood (Saedi et al., 2018). Significantly, our results indicated that the expression of *POMC* in PF was relatively lower than in MF, while *GNRH1*, which has been reported to play a key role in determining sheep litter size (An et al., 2013), displayed a reverse expression pattern between PF and MF. Therefore, we hypothesized that a negative regulatory relationship between POMC and GNRH1 may exist in sheep hypothalamus.

### Functional Analysis of DEGs in PL vs. ML

In functional enrichment analysis of DEGs in PL vs. ML, some pathways including the Jak-STAT signaling pathway (*PRL*, *GH*, *CRLF2*, ENSOARG00000007618, ENSOARG00000016231, and *IL2RB*) were highly enriched. The current study argued that the Jak-STAT signaling pathway in mice was involved in GnRH activities (Ko et al., 2018). *PRL*, as mentioned above, plays an important role in GnRH activities (Araujo-Lopes et al., 2014). The expression of *PRL* was detected not only in the follicular phase but also in the luteal phase, and interestingly, there was a reverse expression pattern of *PRL* between PF vs. MF and PL vs. ML, suggesting its crucial roles in reproduction. The effects of leptin on GnRH release have been revealed (Guzmán et al., 2019), and the infusion of leptin into the arcuate nucleus in rats could cause *PRL* release (Watanobe, 2010), which suggested that *PRL* can be a downstream factor activated by leptin to function in GnRH activities. In addition, the overexpression of growth hormone (GH) could disrupt the state of reproduction, mainly through mediating leptin activities (Chen et al., 2018). Additionally, estrogen could play an inhibitory role on GH *in vivo* (Leung et al., 2003). Collectively, considering the effects of *PRL* and GH on leptin, we speculated that GH, leptin, and *PRL* may coordinate to inhibit GnRH release.

### The Regulatory Network of miRNA–mRNA After Transcription in PF vs. MF

To better understand the functions of miRNAs, a negative interactome containing 5 mRNAs and 4 miRNAs in PF vs. MF was built. Cyclin-dependent kinase 3 (*CDK3*), targeted by Novel_237, was reported that the downregulation of activities of CDK3-related kinase could promote cell apoptosis in the rat (Braun et al., 1998). Immediate early response 3 (*IER3*), targeted by Novel_327, was also involved in enhancing (Zhou et al., 2017) or mediating (Jin et al., 2015) cell apoptosis. Polycystic kidney and hepatic disease gene 1 (*PKHD1*), targeted by Novel_401, has been discovered to induce cell apoptosis, after being downregulated through the PI3K and NF-κB pathways (Sun et al., 2011). Furthermore, our sequencing data indicated that *CDK3* and *IER3* were downregulated while *PKHD1* was upregulated in PF vs. MF. All in all, we hypothesized that more nerve cell apoptosis occurred in MF than PF, which may further influence hormone activities associated with reproduction and may lead to the final observed litter size differences.

### The Regulatory Network of miRNA–mRNA After Transcription in PL vs. ML

The regulatory network of miRNA–mRNA after transcription in PL vs. ML was divided into two main negative networks: the main upregulated and the main downregulated network. In the main upregulated network, thyrotropin-releasing hormone (*TRH*), co-regulated by oar-miR-379-5p, oar-miR-30b, oar-miR-152, oar-miR-495-3p, oar-miR-143, oar-miR-106b, oar-miR-218a, and oar-miR-148a, has been reported to function in GnRH release (see below). Triclosan in mice was found to reduce the production of TRH and thyroid-stimulating hormone (TSH), and this decreased effect could further cause hyperprolactinemia. Hyperprolactinemia was suggested to cause a suppressive effect on kisspeptin expression, resulting in deficits in reproductive and endocrine function (Cao et al., 2018b). In addition, TRH can not only stimulate *PRL* release but also inhibit LH release, and this inhibitory effects may occur through prohibiting the release of GnRH (Araujo-Lopes et al., 2014). Collectively, TRH in the hypothalamus may be responsible, at least in part, for the suppression of GnRH activities.

In the main downregulated network of miRNAs, transthyretin (*TTR*) was reversely regulated by oar-miR-432. The expression level of *TTR*’s in rats could be enhanced by progesterone *via* progesterone receptors both *in vitro* and *in vivo* (Quintela et al., 2011), and a similar upregulated effect of TTR caused by progesterone in mouse uterus was also observed (Diao et al., 2010). Furthermore, TTR could drive the nuclear translocation of insulin-like growth factor 1 receptor (IGF-1R) (Vieira et al., 2015), which could lead to functional changes in insulin-like growth factor 1 (IGF1). Interestingly, the stimulatory effect of IGF1 on GnRH release has been discovered (Hiney et al., 2009). Therefore, we speculated that the negative feedback effects of progesterone on GnRH release may be mediated by TTR, which reduces the binding probability between IGF1 and its receptor, further resulting in a suppression of GnRH activities.

All results indicated that several key DEGs and DE miRNAs in the hypothalamus directly or indirectly participate in hormone activities associated with reproduction, and further studies involving gene/miRNA knockout or overexpression could help us to understand their real functions in female reproductive traits.

## Conclusion

As far as we know, this study provides the first integral mRNA–miRNA interactome in sheep without FecB mutation from the perspective of the hypothalamus. We identified several DEGs (e.g., *POMC*, *GNRH1*, *PRL*, *TRH*, and *TTR*) and mRNA–miRNA pairs (e.g., *TRH* coagulated by oar-miR-379-5p, oar-miR-30b, oar-miR-152, oar-miR-495-3p, oar-miR-143, oar-miR-106b, oar-miR-218a and oar-miR-148a and *PRL* regulated by oar-miR-432) from the RNA-seq data obtained from sheep hypothalamus, which may function through influencing the activities of GnRH. Our results provide novel insights into the prolificacy mechanism of sheep, which may facilitate the discovery of novel major genes and a deeper understanding of female sheep reproduction.

## Data Availability Statement

All the data obtained from RNA-seq has been deposited in the Sequence Read Archive database under the bioproject numbers PRJNA529384 and PRJNA532808.

## Ethics Statement

The animal study was reviewed and approved by the Science Research Department (in charge of animal welfare issues) of the Institute of Animal Sciences, Chinese Academy of Agricultural Sciences (IAS-CAAS) (Beijing, China). Written informed consent was obtained from the owners for the participation of their animals in this study.

## Author Contributions

WH and MC designed the research. ZZ wrote the paper. JT, ZZ, WH, SG, XZ, and JZ collected the data. ZZ performed the study. ZZ and JT analyzed data. MC and WH revised the final manuscript. All authors reviewed the manuscript and approved the final version.

## Funding

This work was supported by the National Natural Science Foundation of China (31501941, 31772580 and 31472078), the Genetically Modified Organisms Breeding Major Program of China (2016ZX08009-003-006 and 2016ZX08010-005-003), the Earmarked Fund for China Agriculture Research System (CARS-38), the Central Public-Interest Scientific Institution Basal Research Fund (2018-YWF-YB-1, Y2017JC24, 2017ywf-zd-13), the Agricultural Science and Technology Innovation Program of China (ASTIP-IAS13, CAAS-XTCX2016010-01-03, CAAS-XTCX2016010-03-03, CAAS-XTCX2016011-02-02), the China Agricultural Scientific Research Outstanding Talents and Their Innovative Teams Program, the China High-level Talents Special Support Plan Scientific and Technological Innovation Leading Talents Program (W02020274), and the Tianjin Agricultural Science and Technology Achievements Transformation and Popularization Program (201704020), Joint Funds of the National Natural Science Foundation of China and the Government of Xinjiang Uygur Autonomous Region of China (U1130302). The APC was funded by the National Natural Science Foundation of China (31772580).

## Conflict of Interest

The authors declare that the research was conducted in the absence of any commercial or financial relationships that could be construed as a potential conflict of interest.
