# Supplementary material for: Integrated Hypothalamic Transcriptome Profiling Reveals the Reproductive Roles of mRNAs and miRNAs in Sheep
Source: Front Genet. 2020 Jan 15;10:1296. doi: 10.3389/fgene.2019.01296 (PMC6974689; doi:10.3389/fgene.2019.01296)
Supplement: Supplementary file 1 [file DataSheet_1.zip › Supplementary Materials/captions.docx]

Supplementary Material

**Supplementary Table S1 |** Real-time quantitative polymerase chain reaction primers and sizes of the amplification products of the selected mRNAs, miRNAs and housekeeping genes.

**Supplementary Table S2 |** Overview of the quality control of mRNA reads generated from hypothalamic tissues.

**Supplementary Table S3 |** The fragments per kilobase per million mapped fragments (FPKM) values of mRNAs identified in polytocous sheep in the follicular phase (PF), polytocous sheep in the luteal phase (PL), monotocous sheep in the follicular phase (MF), and monotocous sheep in the luteal phase (ML).

**Supplementary Table S4 |** The region of identified mRNAs in polytocous sheep in the follicular phase (PF), polytocous sheep in the luteal phase (PL), monotocous sheep in the follicular phase (MF), and monotocous sheep in the luteal phase (ML) from the genome.

**Supplementary Table S5 |** Chromosome distribution of identified genes in the follicular phase (PF), polytocous sheep in the luteal phase (PL), monotocous sheep in the follicular phase (MF), and monotocous sheep in the luteal phase (ML) from the hypothalamus.

**Supplementary Table S6 |** The overall genes expressed in polytocous sheep in the follicular phase versus monotocous sheep in the follicular phase (PF vs. MF) and polytocous sheep in the luteal phase versus monotocous sheep in the luteal phase (PL vs. ML), where the yellow represents differentially expressed genes.

**Supplementary Table S7 |** Overview of the quality control of miRNAs reads generated from the hypothalamic tissues.

**Supplementary Table S8 |** The transcripts per million (TPMs) value of miRNAs identified in polytocous sheep in the follicular phase (PF), polytocous sheep in the luteal phase (PL), monotocous sheep in the follicular phase (MF) and monotocous sheep in the luteal phase (ML).

**Supplementary Table S9 |** Chromosomes distribution of identified miRNAs in the follicular phase (PF), polytocous sheep in the luteal phase (PL), monotocous sheep in the follicular phase (MF), and monotocous sheep in the luteal phase (ML) from the hypothalamus.

**Supplementary Table S10 |** The identification involving diverse RNAs in the follicular phase (PF), polytocous sheep in the luteal phase (PL), monotocous sheep in the follicular phase (MF), and monotocous sheep in the luteal phase (ML).

**Supplementary Table S11 |** The prediction of target genes of miRNAs in polytocous sheep in the follicular phase versus monotocous sheep in the follicular phase (PF vs. MF), and polytocous sheep in the luteal phase versus monotocous sheep in the luteal phase (PL vs. ML).

**Supplementary Table S12 |** The overall miRNAs expressed in polytocous sheep in the follicular phase versus monotocous sheep in the follicular phase (PF vs. MF), and polytocous sheep in the luteal phase versus monotocous sheep in the luteal phase (PL vs. ML), where the yellow represents differentially expressed miRNAs.

**Supplementary Table S13 |** GO enrichment annotation for mRNAs in terms of their molecular function (MF), biological process (BP), and cellular component (CC) level in polytocous sheep in the follicular phase versus monotocous sheep in the follicular phase (PF vs. MF), and polytocous sheep in the luteal phase versus monotocous sheep in the luteal phase (PL vs. ML), where the yellow represents significant enrichment.

**Supplementary Table S14 |** KEGG enrichment annotation for mRNAs in the follicular phase (PF vs. MF), and polytocous sheep in the luteal phase versus monotocous sheep in the luteal phase (PL vs. ML), where the yellow represents significant enrichment.

**Supplementary Table S15 |** The list of target genes of differentially expressed miRNAs in polytocous sheep in the follicular phase versus monotocous sheep in the follicular phase (PF vs. MF), and polytocous sheep in the luteal phase versus monotocous sheep in the luteal phase (PL vs. ML).

**Supplementary Table S16 |** The overlapped gene list between mRNAs and predicted target genes of miRNAs in polytocous sheep in the follicular phase versus monotocous sheep in the follicular phase (PF vs. MF) and polytocous sheep in the luteal phase versus monotocous sheep in the luteal phase (PL vs. ML).
